# Distinct serum biosignatures are associated with different tuberculosis treatment outcomes

**DOI:** 10.1016/j.tube.2019.101859

**Published:** 2019-09

**Authors:** Katharina Ronacher, Novel N. Chegou, Léanie Kleynhans, Joel F. Djoba Siawaya, Nelita du Plessis, André G. Loxton, Elizna Maasdorp, Gerard Tromp, Martin Kidd, Kim Stanley, Magdalena Kriel, Angela Menezes, Andrea Gutschmidt, Gian D. van der Spuy, Robin M. Warren, Reynaldo Dietze, Alphonse Okwera, Bonnie Thiel, John T. Belisle, Jacqueline M. Cliff, W. Henry Boom, John L. Johnson, Paul D. van Helden, Hazel M. Dockrell, Gerhard Walzl

**Affiliations:** a DST-NRF Centre of Excellence for Biomedical Tuberculosis Research, South African Medical Research Council Centre for Tuberculosis Research, Division of Molecular Biology and Human Genetics, Faculty of Medicine and Health Sciences, Stellenbosch University, Cape Town, South Africa; b Mater Research Institute, The University of Queensland, Translational Research Institute, Brisbane, Queensland, Australia; c Specialised Diagnostics and Research Unit, National Public Health Laboratory (URDS/LNSP), Libreville, Gabon; d Centre for Statistical Consultation, Stellenbosch University, Stellenbosch, South Africa; e Núcleo de Doenças Infecciosas, Centro de Ciências da Saúde, Universidade Federal do Espírito Santo, Vitória, Brazil; f Uganda-Case Western Reserve University Research Collaboration, Makerere University College of Health Sciences, Mulago Hospital, Kampala, Uganda; g Tuberculosis Research Unit, Department of Medicine, Case Western Reserve University School of Medicine and University Hospitals Cleveland Medical Center, Cleveland, United States; h Department of Microbiology, Immunology and Pathology, Colorado State University, Fort Collins, CO, USA; i Department of Immunology and Infection, Faculty of Infectious and Tropical Disease, London School of Hygiene and Tropical Medicine, London, United Kingdom

**Keywords:** Tuberculosis, Tuberculosis treatment, Relapse, Biomarkers, Treatment failure, TB, Tuberculosis, TTP, time to positivity, BMI, body mass index, CXR, chest X-rays, RFLP, restriction fragment length polymorphism, TBRU, Tuberculosis Research Unit, IRB, Institutional Review Board, SD, standard deviation, ANOVA, analysis of variance, LOOCV, leave-one-out cross-validation, Dx, diagnosis, M2, month 2

## Abstract

Biomarkers for TB treatment response and outcome are needed. This study characterize changes in immune profiles during TB treatment, define biosignatures associated with treatment outcomes, and explore the feasibility of predictive models for relapse. Seventy-two markers were measured by multiplex cytokine array in serum samples from 78 cured, 12 relapsed and 15 failed treatment patients from South Africa before and during therapy for pulmonary TB. Promising biosignatures were evaluated in a second cohort from Uganda/Brazil consisting of 17 relapse and 23 cured patients. Thirty markers changed significantly with different response patterns during TB treatment in cured patients. The serum biosignature distinguished cured from relapse patients and a combination of two clinical (time to positivity in liquid culture and BMI) and four immunological parameters (TNF-β, sIL-6R, IL-12p40 and IP-10) at diagnosis predicted relapse with a 75% sensitivity (95%CI 0.38–1) and 85% specificity (95%CI 0.75–0.93). This biosignature was validated in an independent Uganda/Brazil cohort correctly classifying relapse patients with 83% (95%CI 0.58–1) sensitivity and 61% (95%CI 0.39–0.83) specificity. A characteristic biosignature with value as predictor of TB relapse was identified. The repeatability and robustness of these biomarkers require further validation in well-characterized cohorts.

## 1 Introduction

Shortening of tuberculosis (TB) treatment would save health care resources, improve adherence and is a main aim of TB drug research. There is evidence that a shortened course of antibiotics is sufficient to cure a large percentage of patients, who respond early to chemotherapy [1] and who has low smear grades or the absence of cavitation at baseline, whereas others require six-month or longer treatment regimens [2]. At this time, it is not possible to implement resource-saving stratified care, as this would require reliable identification of patients with different treatment outcomes and treatment duration requirements.

Currently the best studied biomarker for successful TB treatment is conversion to negative sputum culture at month two of treatment [[3], [4], [5]]. This marker, however, has poor individual predictive ability with a sensitivity of only 40% [6]. High bacterial load at diagnosis as measured by a short time to positivity (TTP) in bacterial culture is associated with risk for relapse [7,8]. The decision to shorten TB treatment based on month two culture conversion and on the absence of cavitary disease on chest radiology alone is not sufficient and results in an increased risk of relapse [9]. Novel biomarkers for TB treatment response and outcome are, therefore, urgently needed. Several studies have investigated individual host immune markers to evaluate treatment response, mostly in small numbers of patients [reviewed in Ref. [10]], but show little promise. Therefore, combinations of immunological, microbiological and clinical predictive markers need to be evaluated.

The current study investigates a large number of host molecules in serum from patients with cured, relapsed or failed TB treatment outcomes. We demonstrate that concentrations of multiple cytokines change significantly during TB treatment, that biological pathways respond with different kinetics and that relapse can be predicted prior to initiation of TB treatment based on a combination of clinical, microbiological and serum immunological markers. These important proof-of-concept findings will lead to new approaches to discover biomarkers of poor TB treatment outcomes and may enable stratification of patients who require shorter or longer treatment regimens.

## 2 Materials and methods

### 2.1 Study participants and sample collection

The study was approved by the Stellenbosch University Human Research Ethics Committee and was conducted according to the Helsinki Declaration and International Conference of Harmonisation guidelines. The present work is a sub-study of the Action TB study, which investigated treatment outcomes in a standard TB treatment trial in Cape Town, South Africa [7]. Seventy-eight cured TB patients, 15 patients with failed month six treatment outcomes and 12 patients who relapsed within two years after initial cure were selected from the 263 trial participants (discovery cohort). All patients were HIV negative, had sputum culture confirmed drug-sensitive TB and adhered to treatment (>80% of dosages observed to be taken). Treatment adherence was monitored by study-specific research nurses who reviewed the pill doses taken. Nurses counted the number of prescribed pills taken and participants were recalled, on the same day, if a dose was missed. The presence of *Mycobacterium tuberculosis* was confirmed in positive cultures with ZN staining and IS6110 DNA fingerprinting and contamination ruled out with blood agar plating. Exclusion criteria were history of previous TB, diabetes, malignancy, pregnancy, chronic bronchitis, sarcoidosis and systemic steroid use. Cure was defined as negative sputum cultures (BACTEC 460 radiometric mycobacterial broth culture system) at month five and six of treatment without recurrent disease during two year follow up, relapse as initial cure at month six but culture proven relapse (with the same strain according to IS6110 DNA fingerprinting when compared to baseline cultures) between three and twenty-four months after end of initial treatment, and failed treatment as positive month six sputum culture with drug sensitive organisms. Clinical information, including body mass index (BMI), was recorded and chest X-rays (CXRs) were obtained at diagnosis.

**Fig. 1:**
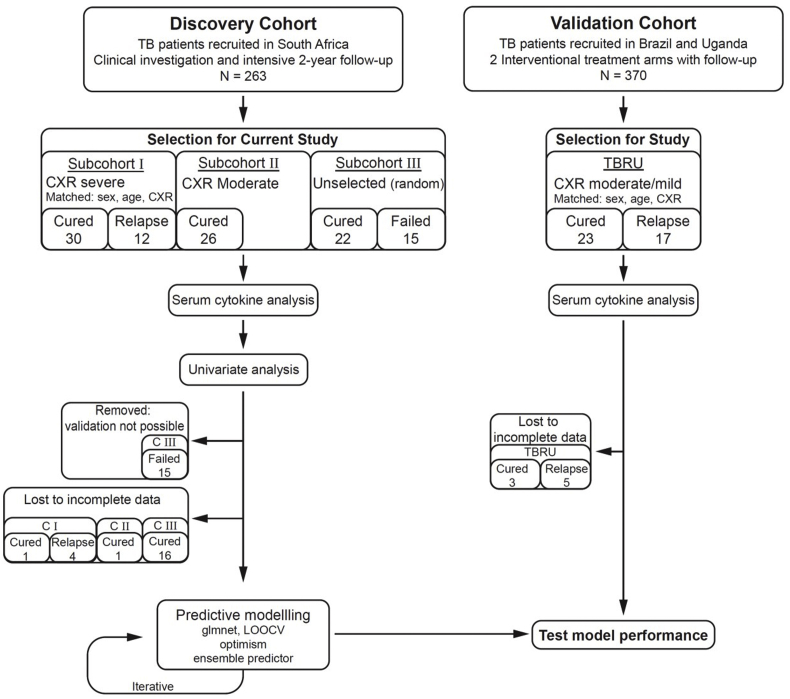
**Overall study design and numbers of patients included in the South African discovery cohort and TBRU cohort.** Patients were selected from the discovery cohort in three phases (subcohort I − III) as funding became available. Of the 263 TB patients recruited in South Africa 12 had documented relapse. Initially these 12 relapse patients were selected and matched according to sex, age and extent of disease on chest X-ray (CXR) at diagnosis (subcohort I). As those patients all had extensive disease, 26 cured patients with moderate extent of disease on CXR were added in phase 2 (subcohort II). Finally, 15 treatment failure patients were added as well as 22 randomly selected cured patients (subcohort III). The prediction models using combined clinical, microbiological and immunological parameters were then applied to the TBRU cohort (validation cohort), which consisted of 17 relapse patients and 23 matched cured patients from Uganda and Brazil.

This study was carried out in three phases. We initially selected 12 relapse patients and 30 cured patients who were matched according to sex, age and extent of disease on CXR (discovery subcohort I, Fig. 1). Two or three matched cured patients were included for each relapse or failure patient. As most of these patients had severe extent of disease, we subsequently added 26 cured patients with moderate extent of disease to have a wider spread across disease severity (discovery subcohort II). Finally, we added the 15 treatment failures and 22 cured patients with both severe and moderate disease (discovery subcohort III). An overview of the participant selection strategy is shown in Fig. 1. Matching of participants into moderate or severe extent of disease on posterior-anterior plain chest radiograph films was performed by two clinicians who were blinded to the final treatment outcome classification. Moderate extent of disease was defined as total area of involvement less than the size of the right upper lobe and two or less cavities, whereas severe extent included all radiographs with more advanced disease. Two clinicians also calculated an extent of disease score for each radiograph according to a standardized revision [11] of a method first published by Ralph et al. [12] that includes a numerical value between 0 and 100 for the percent of abnormal lung parenchyma plus a score of 40 for any visible cavity. Blood and sputum samples were collected at diagnosis (before initiation of TB treatment) as well as at weeks 1, 2, 4, 6 and week 26 (month 6). Serum was obtained by centrifugation of whole blood and stored in aliquots at −80 °C until further analysis. Sputum culture was done using the BACTEC 460 radiometric mycobacterial broth culture system (Becton Dickinson, NJ, USA) and TTP recorded. Mycobacterial drug susceptibility testing for resistance to both first- and second-line drugs was carried out on samples taken prior to treatment initiation as well as at the end of treatment if cultures remained positive. *Mycobacterium tuberculosis* strains were identified by standardized restriction fragment length polymorphism (RFLP) banding patterns after Southern Blot hybridization with an IS6110 probe in all patients with recurrent TB. If the strain pattern was identical between the first and second episodes of TB, the patient was classified as “relapse” patient, whereas different strains resulted in classification of “re-infection”.

The Tuberculosis Research Unit (TBRU) cohort from Uganda and Brazil has been previously described [9] (DMID 01–009; Clinical Trials.gov Identifier NCT00130247) and included two different TB treatment arms: standard six month or shortened four-month treatment. Adults with pulmonary TB were randomized into one of the treatment arms if they had no cavitation on CXR and if their sputum culture converted to negative on solid media after 8 weeks of treatment. The study was halted early due to increased relapse rates in the shorter treatment arm. A subgroup of 17 relapse cases (13 from the shortened and 4 from the standard treatment arm) as well as 23 cured patients, who were matched according to treatment arm, sex, age and extent of disease, were used for the current study (validation cohort). The parent study was approved by the local and national Institutional Review Boards (IRBs) in Uganda and Brazil. All patients gave written informed consent for study participation and the use of their stored samples for future TB research. Serum samples of these patients were collected at diagnosis, at month two and month six, irrespective of treatment arm.

### 2.2 ELISA assays and Luminex analysis

Commercially available ELISA kits were used to measure soluble urokinase plasminogen activator receptor (suPAR; Virogates, Denmark) and neopterin [Immuno-Biological Laboratories (IBL-America), MN, USA] in serum, according to the manufacturers’ instruction. Standards, samples and controls were assayed in duplicate and read on a BioTek microplate reader (BioTek, VT, USA) at a wavelength of 450 nm and a wavelength correction at 650 nm.

We measured concentrations of up to 72 host immune markers in serum using different Linco-plex kits (Millipore, MA, USA) on the Bio-Plex platform (Bio-Rad Laboratories, CA, USA). The Luminex kits comprised: 1) a cytokine/chemokine kit for detection of EGF, eotaxin, FGF-2, FIt-3L, fractalkine, G-CSF, GM-CSF, GRO, IFN-α2, IL-1α, IL-1ra, IL-3, IL-9, IL-12p40, IL-15, IL-17, IP-10, MCP-1, MCP-3/CCL7, MDC/CCL22, MIP-1α, MIP-1β, sCD40L, TGFα, TNF-α, TNF-β, VEGF; 2) a high sensitivity cytokine kit for IL-1β, IL-2, IL-4, IL-5, IL-6, IL-7, IL-8, IL-10, IL-12p70, IL-13, IFN-γ, GM-CSF, TNF-α; 3) a soluble receptor panel for sCD30, sEGFR, sgp130, sIL-1RI, sIL-1RII, sIL-2Rα, sIL-4R, sIL-6R, sRAGE, sTNFRI, sTNFRII, sVEGFR1, sVEGFR2, sVEGFR3; 4) an acute phase protein panel for CRP, Serum Amyloid A (SAP A), Serum Amyloid P (SAP P); 5) matrix metalloproteinase kits for MMP-1, MMP-2, MMP-3, MMP-7, MMP-8, MMP-12 and MMP-13 and 6) a CD8^+^ T cell kit for detection of granzyme A, granzyme B, perforin, sFas/CD95 and sFasL/CD178. GM-CSF and TNF-α were present in both high and low sensitivity kits. However, we report in this manuscript only the GM-CSF and TNF-α concentrations detected with the regular cytokine/chemokine kit. After optimization experiments, samples were diluted (1:8000) with serum matrix (provided by the kit) prior to evaluation with the acute phase protein. For all other kits, the samples were not diluted. All samples were evaluated in duplicate and the quality control values for the different host markers were consistently within the range provided by the manufacturer.

We measured all 72 markers in sera from 30 successfully cured patients, as well as 12 relapse patients (subcohort I in Fig. 1). Subsequently we selected a subset of the most significant markers with respect to changes over the treatment period for the analysis of an additional 48 cured patients as well as 15 failed patients (subcohort II and III in Fig. 1) and 23 cured and 17 relapse patients from the TBRU cohort. *Ex vivo* whole blood of patients from subcohort I has also been used for transcriptomic analysis as published elsewhere [13].

### 2.3 Statistical and pathway analyses

Immune marker values that were too low to be extrapolated by the Bio-Plex Manager Software, version 4.1.1, were imputed to the lowest value that could be extrapolated by the software. Immune marker data were collected incrementally for the subcohorts with fewer markers assayed on successive subcohorts. The immune marker data are typically highly skewed and leptokurtic. We therefore Winsorized values greater than three robust standard deviations (SD) from the robust mean, where the robust statistics were based on 10% trimmed values. The Winsorization procedure retained the order of the extremes instead of censoring to a single fixed value, i.e., values were spread over a narrow range near three SD.

We performed univariate analysis using methods robust to imbalanced design. Predictive modeling required that we trimmed missingness by removing immune markers, and where necessary participants, until the data no longer showed systematic missingness. We imputed missing data using a random forest approach implemented in the *missForest* [14,15] package in the R language and environment for statistical computing software, version 3.4.3 [16]. Data for 18 cured and four relapse participants from the discovery cohort and three cured and five relapse participants from the validation cohort could not be imputed and were excluded from the analysis (Fig. 1).

We compared demographic variables between the groups using one-way analysis of variance (ANOVA) for continuous variables and the Chi-square test for categorical variables. We performed univariate analyses for repeated measures using a mixed-effects linear model as implemented in *lmer* from the *lme4* package [17] with a Tukey HSD *post hoc* test as implemented in *glht* in the *multcomp* package [18]. We used a 5% significance level (p < 0.05) to determine statistical significance.

The pathway analysis were generated through the use of IPA (Qiagen Inc., https://www.qiagenbioinformatics.com/products/ingenuity-pathway-analysis) to determine the canonical pathways in which the clusters of markers play a role.

Prior to predictive modeling, we performed a PCA, which revealed separation of cohorts (batches). We therefore scaled the values by cohort (batch) to diminish batch effects and verified by PCA that the batches were no longer separated. This was necessary since relapse cases were separated in batches and although the separation of batches could have reflected real differences, batch separation could also contribute to inflated performance estimates. We generated predictive models using *glmnet* [19], which is a weighted average of lasso and ridge regression models, each with coefficients for the variables, which are used numerically. A weighting parameter (alpha) determines how to combine the predictions of the two regression models. In class prediction, regression is logistic and the coefficients and weighting parameter can be used to generate a weighted probability of class membership. The training set comprised subcohorts I, II and III and a test set that was the TBRU cohort.

**Supplemental Fig. 1:**
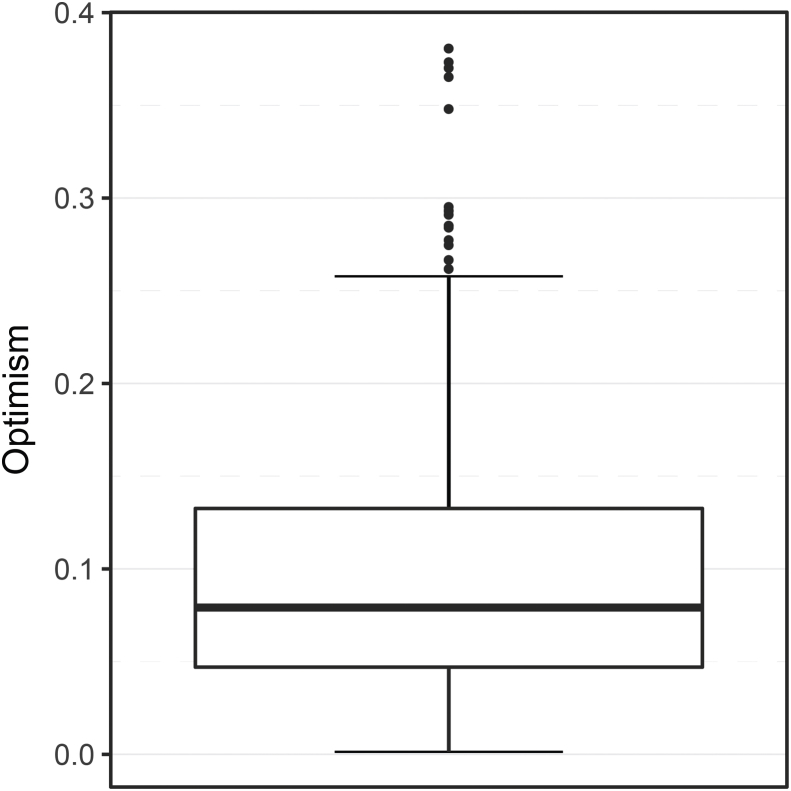
**Distribution of optimism scores for the training set**. We estimated model performance using Harrell's optimism by creating 500 bootstrap (sampling with replacement) iterations of the training set. We performed the full modeling process on the bootstrap training set and predicted on the test set and computed the C statistics (essentially equivalent to the area under the curve) for both. Optimism is defined as C_boot_−C_test_. The ideal optimism is as small as possible.

**Supplemental Fig. 2:**
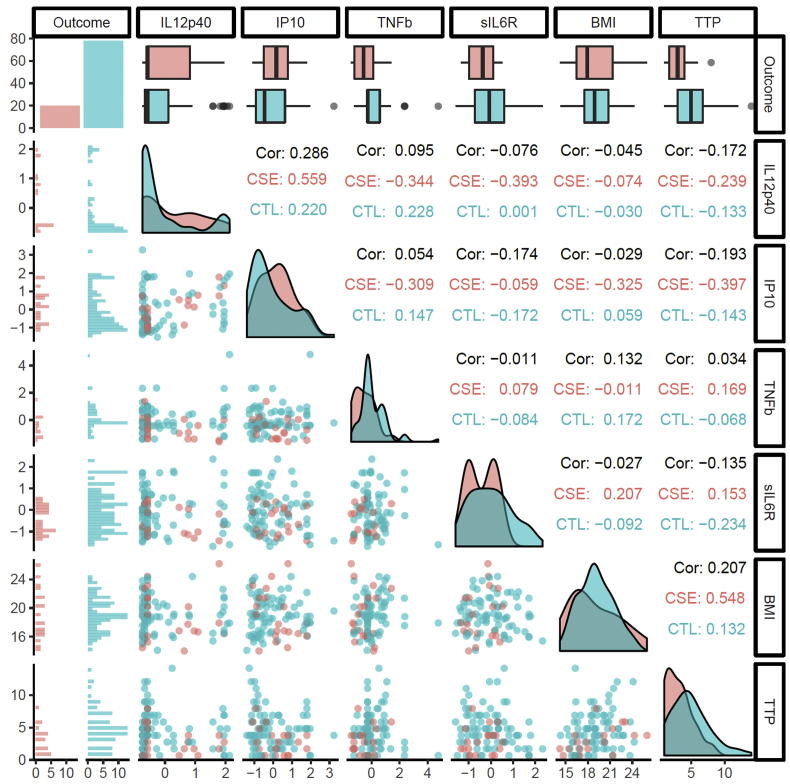
**Relationship between final model variables: Outcome, IL12p40, IP10, TNFb, sIL6R, BMI and TTP**. BMI and TTP are on their original scale, whereas the cytokines have been scaled (Z-transform). The figure produced using the *GGAlly* R package [25] is a matrix showing the pairwise relationship between variables with labels for the column (top) and row (right). The diagonal shows the distributions of the variables as either bar graphs for binary variables (Outcome) or smoothed density plots by Outcome for continuous variables. The first column shows histograms of the continuous variables split by Outcome for each variable. The first row shows box plots by Outcome. The lower triangle (below the diagonal) shows pairwise scatter plots and the upper triangle shows the correlation coefficients: Cor, overall; CSE, relapse case; CTL, cured with no relapse “control”. Colors: coral, relapse; sea green, cured. The complex relationship between variables is illustrated by the scatterplots for BMI, TTP, and sIL6R. For a diagonal line with a negative slope through each of the scatterplots, relapse cases with high BMI and moderately high TTP, sIL6R increases the separation and brings them closer to the other relapse cases. The other variables similarly provide some separation that, weighted appropriately in the *glmnet* model, contribute to the model's discrimination and robustness.

Due to the small size of the training set, we built an ensemble of leave-one-out models and we then applied the ensemble to the test set with the predictive score calculated as the mean of the ensemble predictions. The aim was to predict relapse early during treatment; to compensate for the small number of relapses, we weighted the relapse cases three times the controls, forcing the models to predict cases correctly most of the time. Models were built using leave-one-out cross-validation (LOOCV) on the training set. We estimated modeling performance by Harrell's optimism (PMID 7069920), which is a metric used to determine how likely it is that the modeling process tends to overfit to the training sample and is based on bootstrap resampling from the training set. In this context, use of *glmnet* resulted in a median optimism of 0.079 (using 500 bootstrap iterations; Supplementary Figs. 1 and 2). We used the LOOCV results to rank the most informative variable (BMI, TTP, IL-12p40, IP-10, sIL-6R and TNF-β) and built a final model using these variables and all of the training set. We then validated the final model using the TBRU cohort.

## 3 Results

### 3.1 Patient selection (discovery cohort)

A total of 263 TB patients were recruited in South Africa of whom 211 were cured at month six (week 26). Twelve of the 211 relapsed with the same mycobacterial strain within two years after completion of treatment (5.7% relapse rate). Here, we investigated samples from 78 cured, 12 relapse and 15 treatment failure patients (Discovery cohort; Fig. 1).

### 3.2 Clinical characteristics

**Supplemental Fig. 3:**
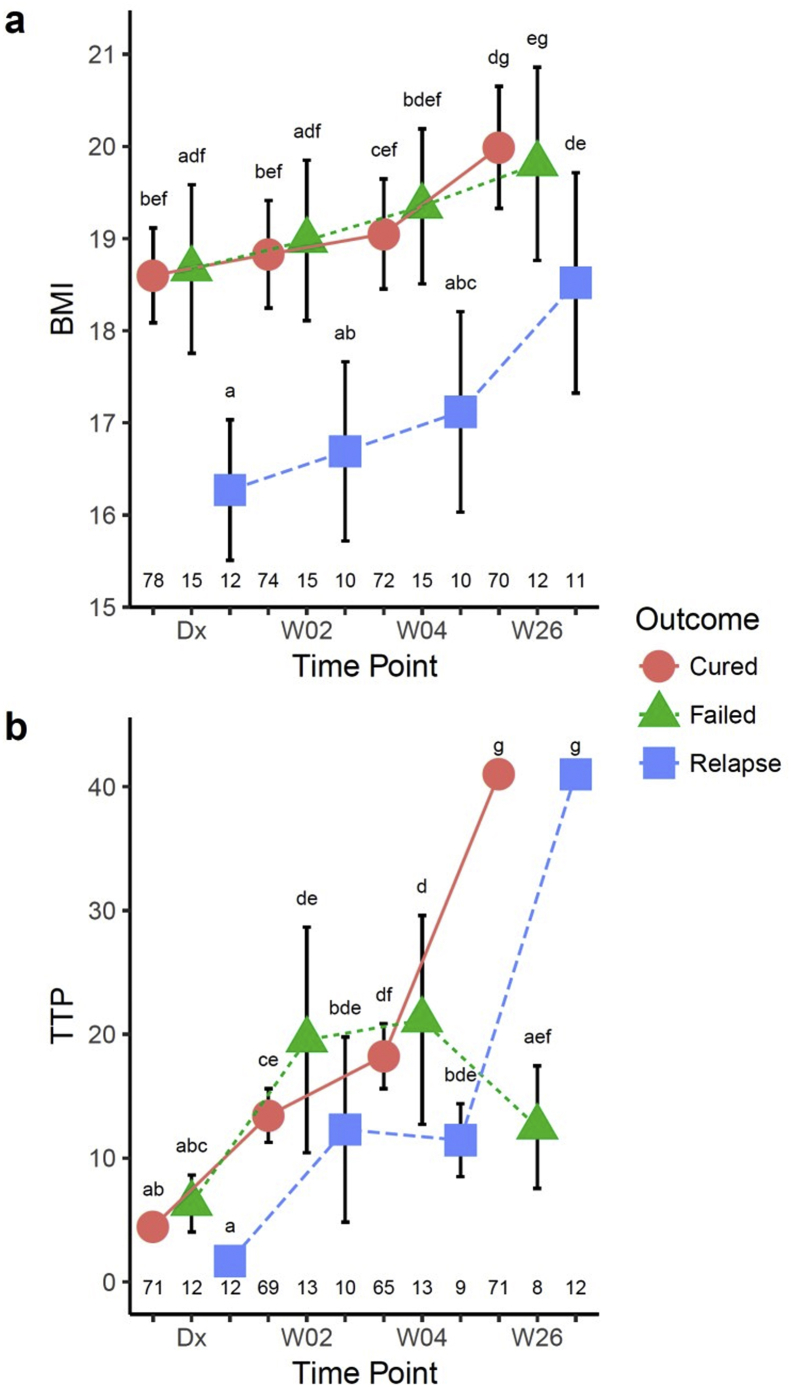
**BMI and TTP of cured, failed and relapse patients.** BMI during the course of TB treatment (a), time to culture positivity (TTP) in days (BACTEC 460 method) during treatment (b) of cured, failed and relapse patients. Data were analyzed by a mixed-effect repeated-measures ANOVA. Each data point represents the mean and the error bars denote the 95% confidence intervals. BMI data are shown as kg/m2 and TTP in days. A TTP of ≥42 is considered as negative. The number of patients in each group at each time-point (n) is indicated above the x-axis. The letters a - g indicate statistical significance where values with the same letter are not significantly different from each other. A p-value of <0.05 was regarded as significantly different.

**Supplemental Fig. 4:**
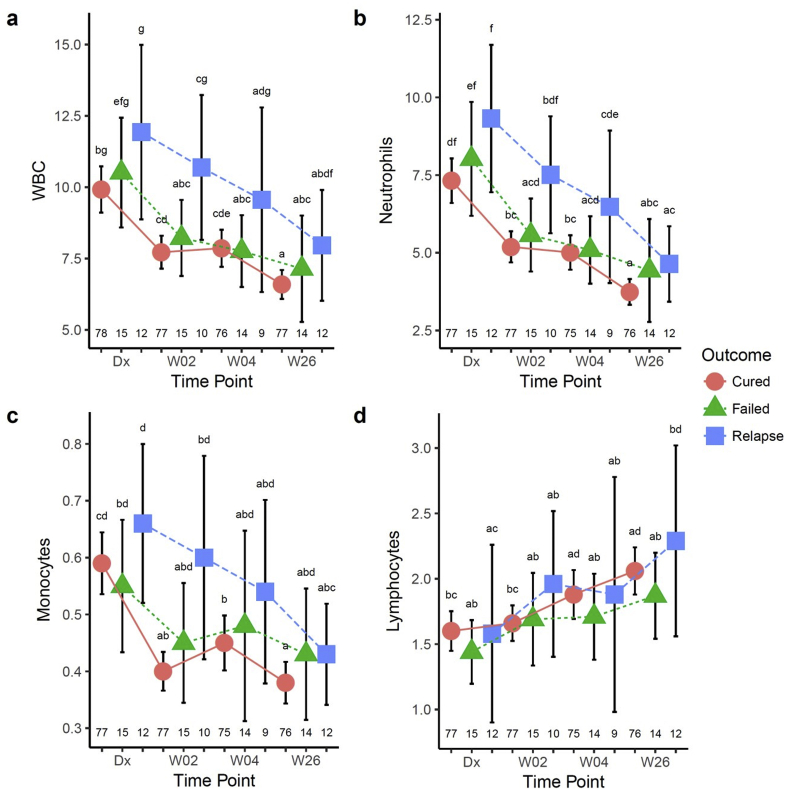
**Differential blood counts in cured, failed and relapse patients during TB treatment.** White blood cell counts (a), absolute numbers of neutrophils (b), monocytes (c) and lymphocytes (c) were determined in cured, failed and relapse patients at diagnosis, weeks 2, 4, and week 26 of treatment and analyzed by a mixed-effect repeated-measures ANOVA. The number of patients in each group at each time-point (n) is indicated above the x-axis. The letters a - g indicate statistical significance where values with the same letter are not significantly different from each other. A p-value of <0.05 was regarded as significantly different.

BMI was significantly lower in patients with subsequent relapse (16.3 ± 1.2, p < 0.01) compared to cured (18.6 ± 2.3) and failed (18.7 ± 1.7) treatment patients at diagnosis and during TB treatment and increased only towards week 26 (Table 1; Supplemental Fig. 3a). TTP at diagnosis was significantly shorter for relapse patients (1.7 ± 0.8 days) compared to the cured (4.5 ± 2.9) and failed (6.3 ± 3.6) treatment outcome groups (Table 1), but statistical significance was lost for TTP over all time points due increased variance (Supplemental Fig. 3b). In addition, TTP was significantly shorter in the failed group at week 26. No association was found between strain genotypes and treatment outcome groups. Extent of disease on CXR at diagnosis was greater in patients with poor outcomes (74.1 ± 31.6 for failed patients and 87.2 ± 25.0 for relapse patients, p < 0.01) compared to cured patients (53.7 ± 32.8; Table 1). Sputum culture conversion at month two occurred in almost half of the participants in the cured (48%) and failed (46%) groups, whereas only 27% of the relapse group converted at month two (Table 1). These findings in the current subset of patients correspond to those reported for the entire Action TB study (6). White blood counts (WBC) and absolute numbers of neutrophils decreased significantly from diagnosis to week two in the cured and failed groups, but not in the relapse group (Supplemental Figs. 4a and b). Monocyte numbers significantly decreased from diagnosis to week two in the cured group only (Supplemental Fig. 4c). WBC, neutrophil and monocyte numbers were significantly lower at the end of treatment in all groups and lymphocyte numbers significantly higher in the cured and relapse groups at the end of treatment (Supplemental Figs. 4a–d).

**Table 1:** Baseline characteristics and month two culture conversion of subsequently cured, failed or relapse TB patients.

Characteristic	Cure (n = 78) n (%) or mean ± SD	Failure (n = 15) n (%) or mean ± SD	Relapse (n = 12) n (%) or mean ± SD	p-value
Sex (Male)	46 (59%)	9 (60%)	9 (75%)	0.57
Age	35.9 ± 10.2^a^	36.7 ± 11.3^a^	41.2 ± 14.0^a^	0.35
BMI (Dx)	18.6 ± 2.3^a^	18.7 ± 1.7^a^	16.3 ± 1.2^b^	<0.01
TTP (Dx)^a^	4.5 ± 2.9^a^	6.3 ± 3.6^a^	1.7 ± 0.8^b^	<0.01
CXR score (Dx)^a^	53.7 ± 32.8^a^	74.1 ± 31.6^ab^	87.2 ± 25.0^b^	<0.01
Month 2 converters^a^	33 (48%)	6 (46%)	3 (27%)	0.44

Different letters in superscript (a,b,c) indicate significant differences between the groups. Letters shared in common between or among the groups indicate no significant difference.

a TTP data at diagnosis (Dx) was not available for 7 cured and 3 failed patients; Chest X-ray (CXR) data was not available for 2 cured, 2 failed and 3 relapse patients; month two (M2) culture data was not available for 9 cured, 2 failed and 1 relapse patient.

### 3.3 Serum immune markers display distinct response patterns during successful TB treatment

**Fig. 2:**
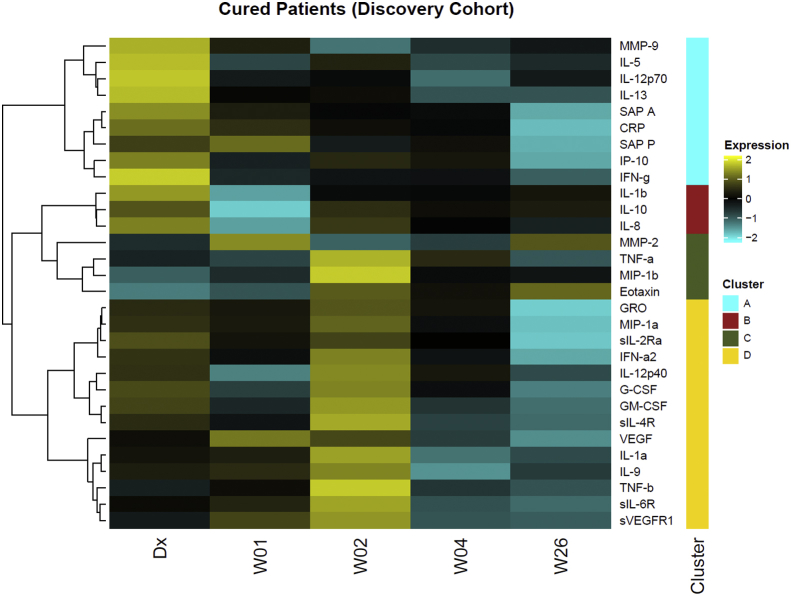
**Cytokine expression patterns during TB treatment in South African cured patients.** Heatmap of serum cytokine levels in 78 cured patients at diagnosis, weeks 1, 2, 4 and 26 of TB treatment. We performed unsupervised hierarchical clustering of 30 markers with Euclidean distance as the metric and the Ward D method of agglomeration, which resulted in four distinct cytokine clusters (A–D). We first log_2_ transformed and centered the data by protein. Bright yellow (+2) indicates 4-fold upregulation from the mean (black) and bright blue (−2) indicates a 4-fold downregulation from the mean. (For interpretation of the references to color in this figure legend, the reader is referred to the Web version of this article.)

We determined the concentrations of 72 immune markers in the serum of a subset of 30 cured and 12 relapse TB patients from subcohort I at initial diagnosis and at weeks 1, 2, 4 and week 26 using Luminex technology. Of the 72 markers evaluated in this study, the concentrations of 30 markers changed during successful TB treatment (data not shown). Subsequently, we assayed the levels of these 30 markers in the remaining patient samples of subcohorts II and III. We performed unsupervised hierarchical clustering of 30 serum analytes from the total of 78 cured patients, which identified four clusters of markers (Fig. 2). Cytokines in cluster A were highest at diagnosis and included inflammatory markers MMP-9, IL-5, IL-12p70, IL-13, SAP A, CRP, SAP P, IP-10 and IFN-γ. Cluster B cytokines were lowest at week 1 and include IL-1β, IL-10 and IL-8. MMP-2, TNF-α, MIP-1β and eotaxin (cluster C) were high at week 1 or week 2 of treatment. Cytokines in cluster D were high at week 2, but low at week 26 and include GRO, MIP-1α, sIL 2-Rα, IFN-α2, IL-12p40, G-CSF, GM-CSF, sIL-4R, VEGF, IL-1α, IL-9, TNF-β, sIL-6R and sVEFGR1. Ingenuity pathway analysis revealed that the unique canonical pathways most strongly associated with the inflammatory markers in each of the clusters are: cluster A, “T helper cell differentiation”; cluster B, “TREM1 signaling and hepatic fibrosis/hepatic stellate cell activation”; cluster C, “granulocyte and agranulocyte adhesion and diapedesis and airway pathology in chronic obstructive pulmonary disease”; and cluster D, “the role of hypercytokinemia/hyperchemokinemia in the pathogenesis of influenza and hematopoiesis from pluripotent stem cells”. Overall, these results indicate that during the 26 weeks of TB treatment, immune modifications occur with different kinetics for different components of the host response.

Patients who fail treatment or who relapse display different serum marker patterns before and during treatment compared to cured patients.

**Fig. 3:**
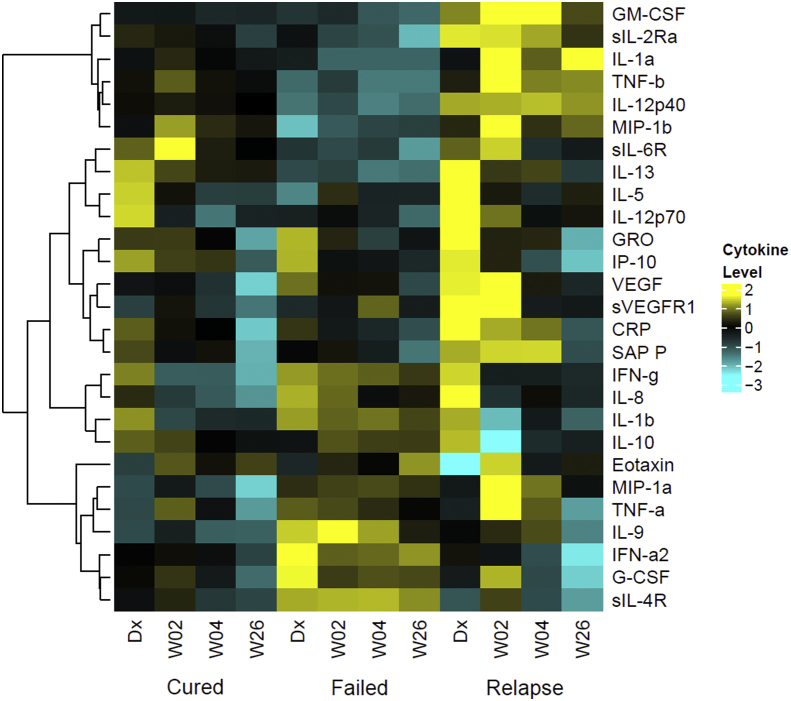
**Cytokine expression patterns during TB treatment in cured, failed and relapsed TB patients in South Africa**. Heatmap of serum cytokine concentrations in 78 cured, 15 failed and 12 relapse patients at diagnosis, weeks 2, 4 and 26 of TB treatment. The heatmap was generated as in Fig. 2. Only cytokines detected in serum of all three patient groups are shown. Bright yellow (+2) indicates 4-fold upregulation from the mean (black) and bright blue (−3) indicates an 8-fold downregulation from the mean. (For interpretation of the references to color in this figure legend, the reader is referred to the Web version of this article.)

Patterns of immune marker expression in the three treatment outcome groups were distinct between cured, failed and relapse patients at the different time points (Fig. 3). Since heatmaps are generated with respect to the mean of the protein expression of the three patient groups, the heatmaps in Fig. 2, Fig. 3 differ subtly in the color shade for the same data. The overall pattern of expression, however, is consistent. Fig. 3 only shows immune markers measured in the serum of each of the three treatment outcomes. Failed treatment patients had lower concentrations of IL-12p40, IL-13, IL-5 and IL-12p70 at diagnosis but elevated concentrations of IL-9, IFN-α, G-CSF and sIL-4R. Relapse patients conversely had multiple cytokines up-regulated at diagnosis, including GRO, VEGF, sVEGFR and CRP as well as during treatment compared to cured or failed patients.

### 3.4 Failed treatment patients have distinct serum cytokine signatures

**Fig. 4:**
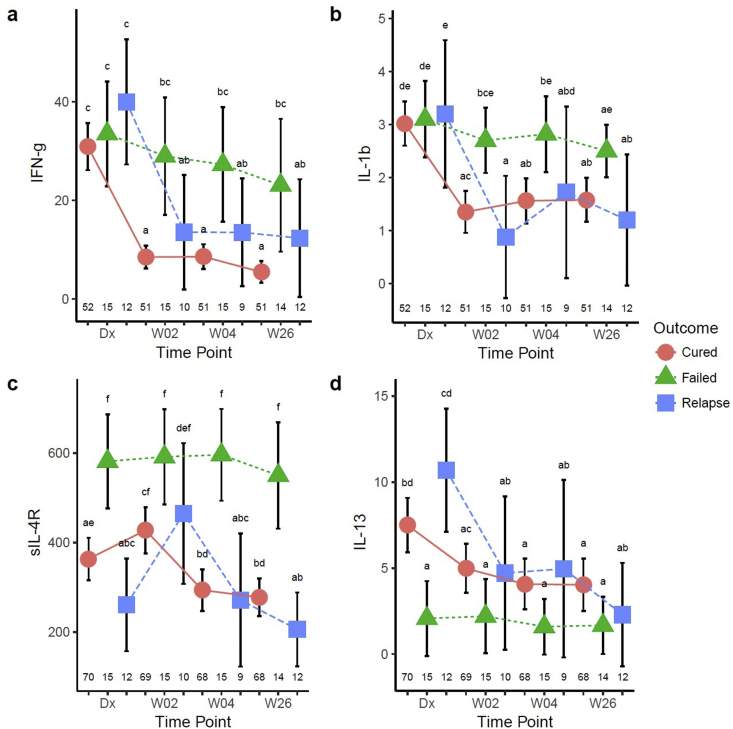
**Patients with failed therapy have different serum cytokine profiles from those with microbiological cure at 6 months (cure and relapse patients) in South Africa.** Serum IFN-γ (a), IL-1β (b), sIL-4R (c) and IL-13 (d) were determined in cured, failed and relapse patients at diagnosis, weeks 2, 4, and 26 of treatment and analyzed by a mixed model repeated measures ANOVA. Each data point represents the mean and the error bars denote the 95% confidence intervals. Cytokine data are shown in pg/ml. The letters a – f indicate statistical significance where values with the same letter are not significantly different from each other. The number of patients in each group at each time-point (n) is indicated above the x-axis. A p-value of <0.05 was regarded as significantly different.

**Supplemental Fig. 5:**
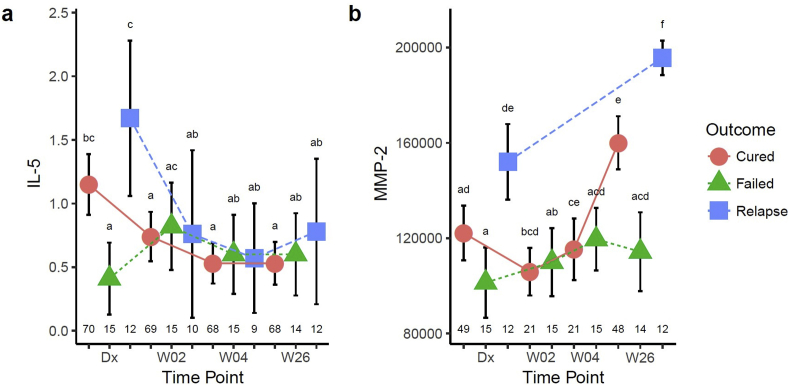
**IL-5 and MMP-2 concentrations in cured, failed and relapse patients.** Serum IL-5 concentrations (pg/ml) (a) were determined in cured, failed and relapse patients at diagnosis, weeks 2, 4, and 26 of treatment and MMP-2 (pg/ml) (b) concentrations at diagnosis weeks 2, 4 and 26 in cured and failed patients and at diagnosis and end of treatment in the relapse patients. Data were analyzed by a mixed-effect repeated-measures ANOVA. The number of patients in each group at each time-point (n) is indicated above the x-axis. The letters a - f indicate statistical significance where values with the same letter are not significantly different from each other. A p-value of <0.05 was regarded as significantly different.

A mixed-effects repeated-measures ANOVA showed that the concentrations of several markers were statistically different in failed treatment patients. The three outcome groups had comparable IFN-γ concentrations at the time of diagnosis, and although cured and relapse cases both had a rapid decline in IFN-γ concentrations within one week, this decline was less pronounced in failed treatment cases (Fig. 4a). A similar pattern was observed for IL-1β (Fig. 4b). Soluble IL-4R concentrations were highest in failed treatment at diagnosis and changed little during treatment (Fig. 4c). Pre-treatment IL-13 (Fig. 4d) and IL-5 concentrations (Supplemental Fig. 5a) were significantly lower in the patients with treatment failure and MMP-2 concentrations remained lowest in these patients at the end of treatment (Supplemental Fig. 5b). Whereas the divergence from treatment responsive cytokine kinetics may reflect ongoing bacterial replication and inflammation in poor outcome groups, the pre-treatment sIL-4R, IL-13, IL-5 and MMP-2 differences suggest that there may be an underlying increased risk of treatment failure in some individuals.

### 3.5 Relapse patients have distinct serum cytokine signatures

**Fig. 5:**
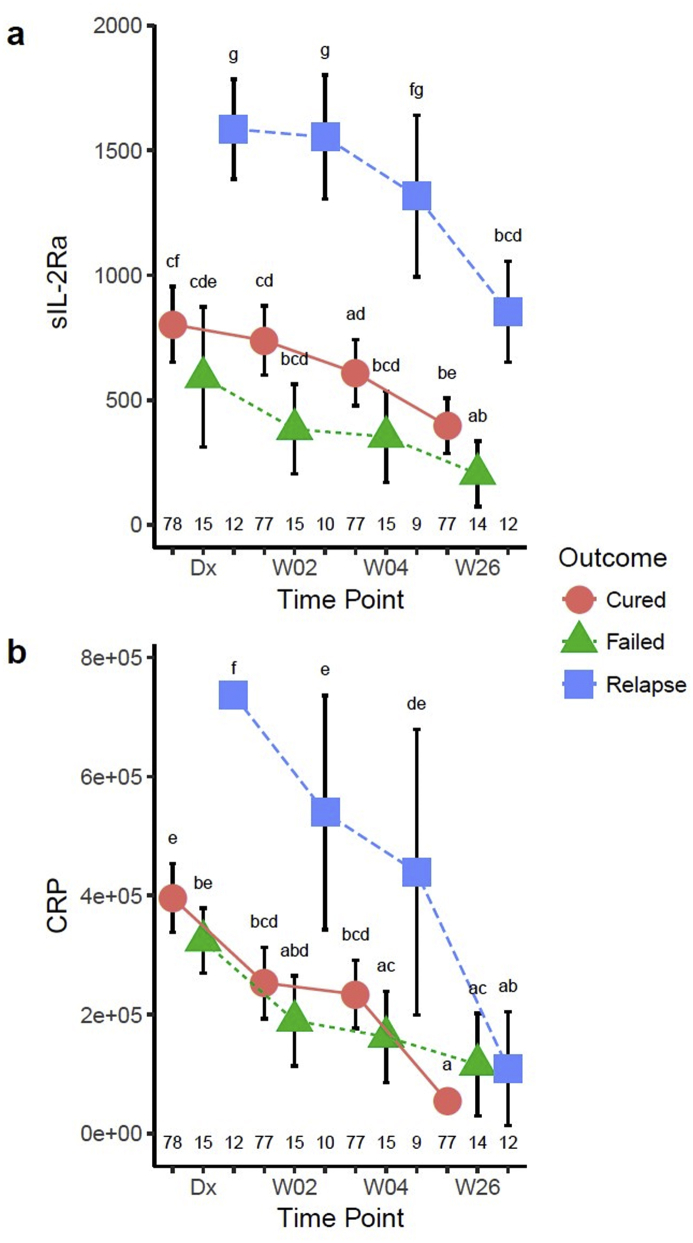
**Cytokines significantly different between relapse and cured/failed patients.** Serum sIL-2R alpha (pg/ml) (a) and CRP (ng/ml) (b) were determined in cured, failed and relapse patients at diagnosis, weeks 2, 4, and 26 of treatment and analyzed by a mixed model repeated measures ANOVA. Each data point represents the mean and the error bars denote the 95% confidence intervals. The letters a – f indicate statistical significance where values with the same letter are not significantly different from each other. The number of patients in each group at each time-point (n) is indicated above the x-axis. A p-value of <0.05 was regarded as significantly different.

Interestingly, sIL-2Rα was highly elevated in relapse patients compared to cured and failed patients at diagnosis and declined only towards the end of treatment (Fig. 5a). Similarly baseline and early treatment concentrations of the acute phase protein CRP were higher in relapse patients but reached similar concentrations to cured and failed patients by the end of treatment (Fig. 5b). The elevated concentrations of acute phase protein in the relapse patients might point towards greater extent of disease, and is consistent with the lower TTP and increased CXR score in this group. Relapse patients also had the highest IL-5 and MMP-2 concentrations at diagnosis (Supplemental Figs. 5a and b). MMP-2 concentrations remained highest at the end of treatment in patients who relapsed (Supplemental Fig. 5b).

### 3.6 A combination of clinical, microbiological and immune markers predicts relapse

**Fig. 6:**
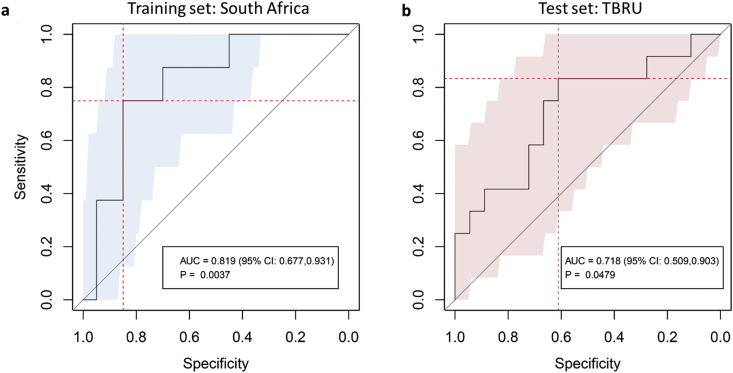
**Receiver operating characteristic (ROC) curve for discriminating relapse patients from cured patients at the start of TB treatment in the discovery/training and validation/test cohorts.** A predictive model was generated using *glmnet* and patients who were recruited in South Africa (discovery cohort) in three phases (subcohort I − III). The predictive model was built using the leave-one-out cross-validation (LOOCV) consisting of 68 individual models (a). The predictive model (average of 68 individual predictions) based on combined clinical, microbiological and immunological parameters was then applied to the TBRU cohort (validation cohort) from Uganda and Brazil (b). Relapse patients can be distinguished from cured patients using six markers measured at diagnosis: BMI, TTP, TNF-β, sIL-6R, IL-12p40 and IP-10 with an area under the curve of 0.819 [95% CI 0.679–0.942] for the training set (a) and 0.718 [95% CI 0.509–0.903] for the validation set (b).

Clinical and microbiological parameters alone are insufficient to predict treatment outcome. Therefore, we hypothesized that a combination of clinical markers together with serum immune markers would increase the accuracy of prediction models. We chose a subset of markers that performed consistently in LOOCV model generation using *glmnet* and found that TTP and BMI with the host markers, TNF-β, sIL-6R, IL-12p40 and IP-10, all measured at diagnosis, could classify relapse patients with a sensitivity of 75% (95% CI: 0.38–1) and specificity of 85% (95% CI: 0.75–0.93) and an AUC of 0.819 (95% CI: 0.677–0.931; p-value = 0.0037) in the training set (Fig. 6a). Since host markers tended toward normal during treatment, models that included host markers not at baseline performed less well. We did not develop a predictive model for failed treatment patients, since we did not have an independent validation set for this group.

### 3.7 Evaluation of the relapse prediction model in an independent cohort

We then assessed whether the models that correctly classified the relapse and cured patients in the South African cohorts would be of value in an independent cohort of patients from different geographical origins. These samples were collected from the participants in a treatment shortening study previously reported by Johnson et al. [9]. The TBRU cohort consists of 17 relapse cases (five from Brazil, of which four were in the shortened four month treatment arm and 12 from Uganda, of which nine were in the shortened treatment arm) as well as 23 cured patients (seven from Brazil, of which six were in the shortened treatment arm and 16 from Uganda, of which 13 were in the shortened treatment arm), which were matched to the relapse cases according to sex and age (Fig. 1). The patients from this TBRU cohort had no cavitation on chest radiograph at diagnosis and all converted their sputum cultures to negative after two months of TB treatment and thus had a lesser radiographic extent of disease compared to the South African cohort. The model described above, consisting of TTP, BMI, TNF-β, sIL-6R, IL-12p40 and IP-10, correctly classified relapse patients from the TBRU cohort with a sensitivity of 83% (95% CI: 0.58–1) and specificity of 61% (95% CI: 0.39–0.83) and an AUC of 0.718 (95% CI: 0.509–0.903; p-value = 0.0479) in this test set (Fig. 6b). The clinical variables (TTP and BMI) alone classified relapse patients from the TBRU cohort with a sensitivity of 33% (95% CI: 0.08–0.58) and a specificity of 100% (95% CI: 1–1) and an AUC of 0.62 (95%CI: 0.39–0.82; p-value = 0.29). The improved performance gained by the addition of immune markers, to clinical parameters, could provide additional prognostic information in treatment outcome studies.

## 4 Discussion

The current study takes advantage of the largest collection of samples from relapse and treatment failure pulmonary TB patients available at present. We describe changes in the serum concentrations of a large number of immune markers during treatment, suggesting that different components of the immune system respond to TB treatment in distinct stages. The study provides promising data that serum immune markers can enhance clinical and bacteriological parameters to improve identification of patients at risk for relapse, enabling stratification of treatment approaches.

Previous studies aiming at prediction of relapse have mainly focused on bacteriological and clinical data. Baseline TTP [8] and month 2 culture status [5] have been associated with risk for relapse but neither test alone is able to reliably predict outcome. Other clinical parameters predisposing TB patients to relapse are low BMI [7] and more extensive disease on chest radiographs at diagnosis, including cavitation and bilateral pulmonary involvement [9,20]. We sought to investigate whether a combination of clinical, microbiological and immunological parameters can increase the prediction of relapse early during treatment. Our study used an integrative approach to identify a six-marker model consisting of TTP, BMI, TNF-β, sIL-6R, IL-12p40 and IP-10 measured at baseline, which predicted relapse with a 75% sensitivity and 85% specificity in the discovery cohort and 83% sensitivity and 61% specificity in the validation cohort. These findings provide important evidence that a combination of clinical, microbiological and immunological markers is superior in predicting poor treatment outcome than clinical parameters alone. Even so, the modest performance of the relapse predicting model in the TBRU samples might illustrate the importance of similar inclusion criteria, such as extent of disease measures and comparable treatment duration in future investigations across different studies, unlike the two studies in the present report.

The results of this study are consistent with the previously reported changes in blood gene expression profiles during TB therapy [[21], [22], [23]]. The rapid down-regulation of 13 inflammatory as well as anti-inflammatory cytokines coincides with the rapid killing of actively dividing bacteria during the early phase of TB treatment. Future research will evaluate whether these rapid cytokine changes could add value to the measurement of early bactericidal activity in clinical trials of novel TB drugs. The ability of host markers that change towards the end of treatment to indicate persistence or clearance of mycobacteria will also have to be evaluated further. Together with the transcriptomic studies on a subset of the same patient cohort [13,23] our results suggest that a comprehensive transformation of the immune response occurs during TB treatment. Furthermore, we show that the characteristic immunologic marker response patterns of curative treatment are different from that of patients with failed TB treatment or relapse. The different kinetics of cytokine changes of patients in the different treatment outcome arms suggests hitherto undiscovered divergent biological mechanisms in each of the outcome types and raises hopes for early prediction of subsequent responses.

Interestingly, some cytokine signatures in the three groups already differ prior to initiation of treatment. The higher concentrations of markers at diagnosis in relapse patients may, at least in part, be attributed to more severe disease as characterized by lower TTP as well as higher chest radiograph score in this cohort. Although the TBRU cohort included only non-cavitary disease, increased total lung radiologic involvement was also noted in relapse patients in that study. The higher concentrations of acute phase markers and matrix metalloproteinases are also in keeping with more extensive disease [24].

The biosignature for failed treatment patients, who did not have greater extent of disease in this study, suggests that their distinct immunological features are due to different mechanisms, which could include host or bacterial genetic as well as environmental factors. The different response patterns of IFN-γ and IL-1β are promising as early markers of treatment failure. Unabated secretion of IFN-γ and IL-1β might be mirroring the continuous presence of *Mycobacterium tuberculosis* antigen.

Despite access to one of the largest available cohorts of relapse and failed patients with samples suitable for biomarker discovery, this study is limited by the low numbers of subjects in the poor outcome groups. Current efforts are under way to establish large sample banks from well-characterized patients on TB treatment and the data presented here will be useful to guide future treatment response biomarker discovery programs. Sample size calculations for future treatment failure biomarker studies with a target of 83% correct classification, indicate that 50 failed treatment patients in both the training and test cohorts would result in a 95% confidence interval of approximately 0.73–0.93%. At a 10% failure rate, this would require 1000 patients. For relapse, assuming that it occurs in in 5% of TB patients, it would be necessary to follow 2000 TB patients for two years post treatment completion to achieve a similar target prediction. Such large studies can only be carried out as multi-site international efforts. In addition, whole genome sequencing of *Mycobacterium tuberculosis* strains would provide a more accurate characterization of relapse cases. The present study, however, provides evidence that combinations of markers hold sufficient promise to justify such an approach.

## 5 Conclusion

This study shows for the first time the breadth of immune system changes as measured through serum markers that occur during TB treatment. Furthermore, different TB treatment outcomes are associated with distinct biosignatures and promising multi-marker signatures that combine clinical, microbiological and immunological markers allow early prediction of treatment failure and relapse. Additional studies with larger sample sizes are required to optimize the combination of host markers for risk stratification and treatment monitoring.

## Funding

The work was supported by the Bill and Melinda Gates Foundation (TB Drug Accelerator Program, grant number 48941); Action TB by GSK; EDCTP (01.T.d1, Grant number 2004.1.R.d1); the South African Technology for Human Resources and Industry Program (THRIP); the Tuberculosis Research Unit at Case Western Reserve University (National Institute of Allergy and Infectious Diseases, National Institutes of Health, grant numbers N01-AI95383 and HHSN266200700022C/N01-AI-70022) and an International Collaborations in Infectious Diseases Research grant from the National Institute of Allergy and Infectious Diseases (grant number 5U01IA115619). This research was also partially funded by the South African government through the South African Medical Research Council, through a grant from the Strategic Health Innovations Partnership (SHIP) unit and by the South African National Research Foundation through a South African Research Chair Initiative: Biomarkers for TB (grant number 86535). The content is solely the responsibility of the authors and does not necessarily represent the official views of the South African Medical Research Council or other funders.

## Conflicts of interest

The authors declare that they have no conflicts of interest.

## Role of funding source

The funding source did not play a role in the study design, collection, analysis or interpretation of the data, or publication of the data.

## Declarations of interest

None.
